# Supplementary material for: Evaluation of the Jichi Medical University diverticular hemorrhage score in the clinical management of acute diverticular bleeding with emergency or elective endoscopy: A pilot study
Source: PLoS One. 2023 Aug 23;18(8):e0289698. doi: 10.1371/journal.pone.0289698 (PMC10446219; doi:10.1371/journal.pone.0289698)
Supplement: Attachment — Submitted filename: Response letter.docx [file pone.0289698.s002.docx]

Thank you very much for reviewing our manuscript entitled “Need for emergency endoscopy in acute colonic diverticular bleeding: a multicenter validation of the JD score.” (PONE-D-22-25709, The title was corrected). We are grateful for the editor’s and reviewers’ thoughtful and valuable comments. We have corrected and revised our manuscript intensively in accordance with them. Data presentation in tables was changed from mean ± SD to median (min-max). We changed the method of statistical analysis from Student’s t-test to Mann-Whitney U test, because a group of patients was not based on a normal distribution. We have also addressed the issues raised by the reviewers in a point-by-point fashion, as outlined below. Red text in the main manuscript indicates the revised portions. We believe that these changes have greatly improved the quality and significance of our study and this version is now suitable for publication in PLOS ONE.

Reviewer #1:

Unfortunately it is not very clear what was the aim of this research and accordingly, the design they used to state their hypothesis.

E.g. in the abstract the authors stated that the aim of this study was "to establish an efficient and safe emergency endoscopy system", what it is neither clear, nor a scientific question. In the introduction they stated that the aim of this study was "to validate our scoring system" (i.e. JD score). The sentence before, the authors stated that "no study to date has examined whether emergency endoscopy should be performed for colonic diverticular bleeding using a scoring system". So, the hypothesis should be something challenging the indication of an emergency endoscopy, but the primary outcome was the identification of the bleeding source, which alone would have no impact. Why not using e.g. endoscopic hemostasis instead? This would be much more interesting. I read in the discussion first, that every patient in the emergency endoscopy group received endoscopic treatment, but exactly numbers and proportions in the elective endoscopy group are not given (which of course should be listed in the results).

**Answer:** 　Thank you for your valuable and important comments.

The aim of this study is to validate the usefulness of JD score, which indicates whether an emergency or elective endoscopy should be performed for colonic diverticular bleeding. Hemostatic treatment has been shown to improve patient outcomes and it requires the identification of the bleeding source in diverticular hemorrhage. Therefore, "bleeding source identification" was set as a primary outcome in this study and it is almost the same as hemostatic treatment. (Page 10, Line164-167)　Regarding the hemostatic treatment in the elective group, the bleeding source was identified in 11/47 (23%) and the hemostatic success rate was 11/11 (100%). we described these in “Results” section (Page 14, Line 230-237) and in Table 2.

E.g. the article could be something like "Need for endoscopic treatment in acute colonic diverticular bleeding: a multicenter validation of the JD score". It would be however questionable to change the design of an intervention trial.

**Answer:** 　Thank you very much for pointing out an important issue. In accordance with your advice,　we corrected　the title.

Further, the JD score is based on CT scan findings, which is however not recommended for every patient with suspected diverticular bleeding Finally, 91% of the patients in the "emergency group" had an extravasation in the CT scan, what would of course increase the probability to see something in the endoscopy (what is the importance of the others parameters in the JD score?). The question of which patients need a CT scan remain open, unfortunately limiting the significance of this study.

**Answer:** 　Contrast-enhanced CT (CE-CT) is an important component and requirement for the JD score. Although there are some arguments concerning the clinical significance of CE-CT in patients with lower gastrointestinal bleeding, its usefulness has been reported in several studies (ref #41, #42, #43 in the manuscript). So, if there are no contraindications, such as renal dysfunction or allergy to contrast agent, we believe it acceptable to perform CE-CT in almost all cases. CE-CT sometimes detect some other disease, leading to avoid emergency endoscopy.

There were only three cases without an extravasation in CE-CT in the emergency group. The other parameters in the JD score may have low significance in this scoring system. However, as shown in Figure 2, higher JD score were commensurate with a higher bleeding source identification rate. Therefore, these parameters help increase the pre-test probability of identifying the bleeding source. We added these descriptions in Page 20, Line 312-319. As you indicate, the question of which patients need a CT scan remain open. We added this point in the limitation of the study　in Page 23, Line 368-373.

Other comments

- methods: "to evaluate the benefit of hemostatic treatment, the patients were divided into those in whom the bleeding source could be identified (identified group) and those in whom it could not (non-identified group)" -> this is not clear, because identification of the bleeding source does not necessarily implicate hemostatic treatment. Additionally, there is no information about hemostatic treatment.

**Answer:** 　All cases with an identifiable source of bleeding were treated with hemostasis in this study. We also described about hemostatic treatment. We added these descriptions in “Materials and Methods” (Page 9, Line 156-161; Page 10, Line 164-167).

- an important number of patients received blood transfusions; however in the demographics (i.e. at least at presentation) none of the patient was unstable or with low hemoglobin. The authors should give more information about hemodynamic stability or low hemoglobin values in the course. It would be also interesting to know which patients needed ICU care.

**Answer:** 　In acute hemorrhage, Hb often does not drop much at the time of presentation. We added shock index to “Table 1” as an indicator of the patient's hemodynamic stability. However, the indicator does not always indicate the severity of the patient's illness and no patient required ICU admission in this study (Page 15, Line 254-255). This may be due to appropriate post-arrival management, hemostasis procedures, blood transfusions, etc.

- the power analysis is not clear. The authors should give more information. Additionally, the authors calculated 60 patients, but recruited 91 (?)

**Answer:** 　The minimum sample size for this study was calculated as 60 and we set the study period long enough to accumulate these patient numbers. During that period, we have collected as many cases as possible to assure a more accurate evaluation. We added this description in Page 11, Line 184-187.

- the authors should give a reference for the comorbidity index

**Answer:** 　We added the reference (ref. #33).

- results without importance (e.g. minimally statistically differences in platelets count or diastolic pressure) should not be reported in the text (is instead OK in the table)

**Answer:** 　We changed the method of statistical analysis from Student’s t-test to Mann-Whitney U test, because a group of patients was not based on a normal distribution. Then, statistical differences in platelets count and diastolic pressure disappeared. So, we deleted these descriptions in “Results” section.

- it would be interesting to know if anticoagulant reversal was performed in these patients

**Answer:** 　Anticoagulant reversal was performed in only one of the three patients in the elective group and none in the emergency group. We added this description column to Table 2 and Table 3.

- how did the authors assess re-bleeding? There was a follow-up? How was performed?

**Answer:** 　Rebleeding was defined as fresh bloody stools after endoscopy, which confirmed hemostasis, or bloody stools with a decrease of more than 2 g/dL of Hb. We added this description in Page 9, Line 161-162.

- the limitations section should be expanded

**Answer:** 　We added the descriptions in the limitation section, concerning the contraindications and reading skills of CT scan, and endoscopist’s skill (Page 23, Line 368-373)

- statements in the discussion e.g. by platelets and blood pressure are overreaching; additionally the authors should not repeat the results but stick to a global interpretation before assessing for external validity

**Answer:** 　We removed duplicate content in “Results” and “Discussion” sections. We revised the “Discussion” section considerably.

- in the discussion the authors should compare endoscopic treatment vs endovascular treatment (especially when an extravasation is seen on the CT scan)

**Answer:** 　When we identified the source of bleeding, we initially applied clipping in all cases, though hemostatic procedure was selected at the discretion of endoscopists. In only one case, hemostasis was not achieved by clipping, and IVR was successfully performed. Therefore, we could not compare endoscopic treatment and any other treatment in this study. Then, we discussed IVR in “Discussion” section (Page 22-23, Line 350-362).

Reviewer #2:

1. I advise authors, when submitting a manuscript to a journal to always include the continuous line number so that reviewers can make a timely and accurate review.

2. For manuscript review, I entered the count on the submitted manuscript without making any other changes.

**Answer:** 　According to your advice, we added line numbers in the manuscript.

References

3. I advise authors to set the style of references as required by the journal. Perhaps using a computer system reference management tool so that it is done properly. https://journals.plos.org/plosone/s/submission-guidelines#loc-references

**Answer:** 　We used “EndoNote” to properly fix the reference style.

Title

4. I advise authors to edit the title.

Since, after reading the manuscript completely I understood that you wanted to do a validation study regarding the previously developed JD score so it would be important that the title also highlight that this is a validation study.

**Answer:** 　Thank you very much for your advice. We corrected the title.

Keywords

5. I advise the authors to double-check through the MESH batabase the keywords:

a. colonic diverticular bleeding does not exist, however, Diverticulum Colon; Bleeding; Diverticular Diseases is present

b. scoring system does not exist, there is score; scoring;

c. emergency endoscopy does not exist, there is however: endoscopy; Endoscopy, Digestive System; Endoscopy, Gastrointestinal;

**Answer:** 　According to your advice, we corrected the key words

Introduction

6. Line 37: I suggest to the authors, as they included reference to the Japanese Gastroenterological Associatio guideline they should also include that of the American Gastroenterological Association (Strate LL, Gralnek IM. ACG Clinical Guideline: Management of Patients With Acute Lower Gastrointestinal Bleeding. Am J Gastroenterol. 2016 Apr;111(4):459-74. doi: 10.1038/ajg.2016.41. Epub 2016 Mar 1. Erratum in: Am J Gastroenterol. 2016 May;111(5):755. PMID: 26925883; PMCID: PMC5099081.) I'm not absolutely sure if this is the very latest version but it seems to be.

**Answer:** 　We added the guideline of American Gastroenterological Association as a reference (ref. #10). We also added the guideline of the European Society of Gastrointestinal Endoscopy (ref. #9).

7. Line 38-39 Is this statement an inference by the authors or is it supported by the low evidence in the guidelines? I suggest the authors specify this better, perhaps putting the guideline passage with what it states and the level of evidence.

**Answer:** 　As you pointed out, this statement is supported by the low level of evidence in the guidelines. We added this description in Page 4, Line 58-59

8. Line 64. I suggest the authors move the written references or add more in correlation to the statement made about "and size of the exposed blood vessel, whether the source of bleeding is an artery or vein, and the location of the diverticulum (visibility)." If these conditions are always integrated into the previously cited references I recommend citing them again, otherwise it seems like a statement by the authors themselves, without a rationale.

**Answer:** 　Thank you very much for pointing out. We corrected the content to be based on the literature and added the reference numbers properly. (Page 5, Line 83-89)

9. Line 64-67 I advise the authors to include references about it here as well or modify the sentence so that it is understood to be a statement by the authors.

**Answer:** 　This was our statement. We think it inappropriate to describe our statement in “Introduction”. Then, we deleted the description and modified wording. (Page 5, Line 83-89)

10. Line 75. The score states that 1 point is taken for the use of oral anticoagulants. It would be important to perhaps specify which types, whether any type of anticoagulant or a particular type.

**Answer:** 　The types of oral anticoagulant, such as DOAC and warfarin, were not specified in this study. We added “any type” in the “Abstract” (Page 2, Line 29) and “Materials and Methods” (Page 7, Line 124).

Materials and methods

11. Line 96-97 Why do the authors in point 2 of the exclusion criteria include "one or more episodes of bloody stools in the previous 30 days" They should justify why this is so.

**Answer:** 　Bleeding within 30 days may indicate a rebleeding. We excluded rebleeding cases, because the effects of previous bleeding would remain, and it may affect the future rebleeding. Although this is our speculation, we defined this as one of the exclusion criteria for this study. In fact, no cases were excluded with this criterion. We added this description in Page 7, Line113-115.

12. Also at line 98 item 4 of the exclusion criteria what do the authors mean by "could not undergo endoscopy at the time indicated in the score." In what way, why could patients not undergo endoscopy? It would be better to justify

**Answer:** 　As shown in Figure 1, two patients in JD score ≥3 did not undergo emergency endoscopy and were treated conservatively at the physician's discretion. Three patients in JD score <3 underwent emergency endoscopy at the discretion of their physicians due to a large amount of blood in the stool or unstable vital signs. We described these situations in “Results” section (Page 12, Line 202-208).

13. I would advise the authors to restructure the materials and methods with subchapters so as to make the work easier and more readable, for example with subchapters such as: inclusion and exclusion criteria, a subchapter with the JD score, perhaps tightening the part in the introduction and expanding the part in the methods, not forgetting that there is already a manuscript about it, so it should not be repetitive but quickly explanatory. I hope I have explained myself. One last subchapter with the two groups of patients, elective and emergency endoscopy.

**Answer:** 　Thank you very much for pointing-out an important issue. According to your advice, we restructured “Materials and Methods” and “Results” sections with subchapters. We think these changes have made our study easier and more readable.

14. With related subgroup developments. This would make the work more linear and logical and easily understood perhaps even by those not fully in the profession.

**Answer:** 　With a subchapter of “Subgroup analysis” in “Results” section, we think it became easy to read (Page 16-17, Line 264-274). As mentioned before, we changed the method of statistical analysis (Page 11, Line188-189). Then, it is found that the identified group had a significantly shorter hospital stay than the unidentified group (Page 17, Line 273-274).

15. Another piece of advice I would give to the authors is to explain whether the election group was being "prepped" with a purgative in order to better assess the bowel or was it conservative without using laxatives for bowel cleansing. Because in Table 2 it is understood that 85% of the elective patients were bowel-prepared but it is not stated in the methods.

**Answer:** 　We explained about bowel preparation in the “Materials and Methods” (Page 8-9, Line 138-146) .

Discussion

16. Line 209-279 The authors make a long digression of the results obtained but unfortunately list the results of the study again in a descriptive manner without going into the results and data obtained but especially not making a comparison with the present literature.

17. The discussion should be a deepening of the results by comparing them with the present, or even past, literature, comparing their own results with those present, perhaps criticizing or pointing out the differences. The fact that no score is present does not mean that one cannot

compare the results obtained, perhaps just with primary endpoint and then the secondary ones.

**Answer:** 　Thank you very much again for pointing-out an important issue. We corrected the entire ”Discussion” section with subchapters. We eliminated duplicates with "Results" section and compared our results with the present literature.

18. How is endoscopy time in the literature? bleeding after urgent or elective endoscopy? Or the days of hospitalization? Do they change from the literature? Transfusions?

**Answer:** 　There was no coherent paper on endoscopic procedure time, and it was only mentioned briefly in some papers. The endoscopic procedure time tended to be longer in our study, compared to other studies. This may be due to our willingness to identify the source of bleeding. Although many papers have found no significant changes in clinical outcomes in emergency endoscopy, patient backgrounds and results differ significantly from paper to paper, making simple comparisons difficult. We added this description in Page 22, Line 342-347.

19. I recommend that the authors remerge in the discussion and elaborate on the various points, not re-describing the results but elaborating on them.

**Answer:** 　As in the response in 16 and 17, we have revised the “Discussion” section in its entirety.

Tables

20. I suggest the authors create legends for the tables, each table a title and an explanatory legend, not meaning to describe the results but to specify acronyms or data presentation and whatnot.

**Answer:** 　Thank you very much for pointing out. We modified the tables and created legends, as you indicated.
