# Supplementary material for: Evaluation of the Jichi Medical University diverticular hemorrhage score in the clinical management of acute diverticular bleeding with emergency or elective endoscopy: A pilot study
Source: PLoS One. 2023 Aug 23;18(8):e0289698. doi: 10.1371/journal.pone.0289698 (PMC10446219; doi:10.1371/journal.pone.0289698)
Supplement: Attachment — Submitted filename: Response letter050523.docx [file pone.0289698.s003.docx]

Thank you very much for reviewing our manuscript entitled “Need for emergency endoscopy in acute colonic diverticular bleeding: a multicenter validation of the JD score.” (PONE-D-22-25709)

We are grateful for the editor’s and reviewers’ thoughtful and valuable comments. As pointed out, this was not a validation study. We should treat this study as a pilot study and have changed the title. We have corrected and revised our manuscript intensively in accordance with your comments.

We believe that these changes have greatly improved the quality and significance of our study and this version is now suitable for publication in PLOS ONE.

Editor’s comments:

a) in M&M it is necessary to immediately clarify the Multicenter study; the two hospitals mentioned must be framed as dimensions (Primary Care, Tertiary Care, etc...) and it is necessary to know approximately how many endoscopists were involved (one per centre? more endoscopists per centre? etc...).

**Answer:** 　Thank you for your valuable comments. We immediately clarified in M&M that this is a prospective multicenter study (Page 6, Line 107). We have added the information about dimensions and the number of endoscopists per center in Page 6-7, Line 109-112.

b) the allocation is never clearly explained, which is only apparent from Figure 1. It must be clearly explained that based on the JD score, it is determined how to treat patients (elective vs urgent). This point is fundamental.

**Answer:** 　 Thank you very much for your valuable and important comments. In the section of “Allocation of patients according to the JD score” in M&M, we clearly explained the flow of patients and the way of grouping according to the JD score (Page 8, Line 131-146). We have also modified Figure 1 to better reflect the actual clinical flow.

c) There are patients who are excluded because they are then managed differently from what was initially planned. They must be reported in absolute value (as has been done), but also in percentage. This shows the limitations of the JD score, which will perhaps require further study in the future with additional parameters to avoid having these exclusions which are significant in percentage terms.

**Answer:** 　Thank you very much for your important comments. We have added the percentage of patients excluded in addition to the absolute value (Page 13, Line 223-225). Since the JD score was designed for diverticular hemorrhage, we excluded cases other than diverticular hemorrhage in order to ascertain the effect of the therapeutic intervention on the score (Page 8, Line 143-144). However, it was unclear in real clinical practice whether lower GI bleeding was due to diverticular bleeding before performing lower GI endoscopy. Therefore, it is important to know whether emergency endoscopy should be performed in patients with lower GI bleeding. It is worthwhile to evaluate the usefulness of JD score with other parameters in patients with lower GI bleeding in the future. Also, we need to extend our study to patients without CE-CT and to evaluate the need of CE-CT in clinical settings. Thank you very much for showing us the future directions.

d) What reviewer #2 pointed out is fundamental and should be placed as a clear limitation in the methodology

**Answer:** 　Thank you very much for your valuable comment. As you noted, this study has many limitations. We added several limitations to show a clear limitation in the methodology（Page 24　Line 384-393）

e) The Authors should reflect on whether it would not be better to present this paper as a Pilot Study, even in the title. The numbers are objectively low and it would be better to call it that.

**Answer:**

As you and reviewer #1 pointed out, this study did not meet validation studies. This study should be treated as a pilot study. So, we changed our title to “Evaluation of the Jichi Medical University diverticular hemorrhage score in the clinical management of acute diverticular bleeding using endoscopy: **a pilot study.**”

I find the paper interesting, but there is a lack of methodological clarity in the exposition. I would therefore ask the authors to tidy up the M&M session, put this part in order as if it were the first time we were re-reading this paper - explaining well each step in the: 1. selection of patients (inclusion/exclusion), 2. allocation of patients according to JD, 3. taking care of patients medically (crystalloids, transfusions, antibiotics, etc.) and 4. when you are further excluded. Because it is unclear to understand the pathway. Consequently, the presentation of the results must also be ordered as in M&Ms, and the limits must be adjusted.

**Answer:** Thank you very much for your important comments. As you indicated, we have restructured the M&M session. We have created several paragraphs to make the text more readable and understandable. We also revised the Result, Discussion, Limitations, and Conclusion sections.

6. Review Comments to the Author

Reviewer #1: Thank you to the authors to resubmit a revised version of the manuscript. Unfortunately there is a major design concern, which appears not possible to address. The JD score was developed to predict the endoscopic identification of the bleeding source in patients with suspected colonic diverticular bleeding. A validation study should accordingly prospectively confirm that patients with a JD score ≥ 3 have an higher endoscopic identification rate of the bleeding source in patients with suspected colonic diverticular bleeding (compared with JD score < 3). This means that each patient should undergo endoscopy and categorized as JD score ≥ 3 (or JD score < 3), and identification of the bleeding source (yes/no). However, at least two patients (with JD score ≥ 3) did not undergo emergency endoscopy and were treated conservatively at the physician's discretion. A physician-based decision in a validation study is however not acceptable. Further, the authors divided patients in an emergency endoscopy vs elective endoscopy group. This seems to refer to another outcome than the identification of the bleeding source (e.g. identification of patients who need emergency endoscopy). If so (which again is not validation of the score), the authors should state another hypothesis and especially another power calculation. Additionally, each patient with a JD score ≥ 3 should undergo emergency endoscopy, and each patient with a JD score < 3 should not. Both of these conditions were not applied to this study.

**Answer:** 　Thank you very much for your valuable and important comments. As you indicated, this study did not meet validation studies. This study should be treated as a pilot study. We corrected the title and revised the main text intensively.

Since the JD score was designed for diverticular hemorrhage, we excluded cases other than diverticular hemorrhage in order to ascertain the effect of the therapeutic intervention on the score (Page 8, Line 136-146). The main objective of this trial is to reduce the number of emergency endoscopies without disadvantage to patients and to reduce the burden on patients and medical staff. However, since these items are difficult to quantify, we hypothesized that patients with a high probability of identifying the bleeding source should undergo emergency endoscopy, and patients with a low probability do not need to undergo emergency endoscopy, but elective endoscopy. Therefore, the bleeding source identification rate, leading to the hemostatic procedure, was set as the primary endpoint (Page10; Line 171-178).

We performed power calculation based on the bleeding source identification rate in our previous study. We apologize for the error in the power calculation. The sample size was recalculated, and the sample size required to detect a significant difference in the emergency endoscopy was 27, not 15 (Page 11; Line190-198). In this study, patients with a JD score <3 were assigned to receive elective endoscopy. With the extrapolation of the result of the previous study, the total required sample size was calculated to be 81. We corrected the number and revised the descriptions in “Statistical analysis” of M&M section.

Reviewer #2: Certainly an adequate level of review has been carried out by the authors.
The work certainly appears to be much more supported by scientific evidence and evidence also present in the literature, although weak, but stated by the authors with a less speculative development of the work and the results obtained by relying solely on the present results.

**Answer:** 　Thank you very much for your valuable and important comments. As you pointed out, this study did not meet validation studies. This study should be treated as a pilot study. We corrected the title and revised the main manuscript intensively. We revised the “Limitations” section to show a clear limitation in the methodology. Further studies are needed to validate out results and this study should be extended to the field of acute lower GI bleeding and cases without CE-CT. We also need to evaluate the indication of CE-CT in real clinical settings.
