# Supplementary material for: Evaluation of the Jichi Medical University diverticular hemorrhage score in the clinical management of acute diverticular bleeding with emergency or elective endoscopy: A pilot study
Source: PLoS One. 2023 Aug 23;18(8):e0289698. doi: 10.1371/journal.pone.0289698 (PMC10446219; doi:10.1371/journal.pone.0289698)
Supplement: Attachment — Submitted filename: Response letter to Reviewer.docx [file pone.0289698.s004.docx]

Review Comments to the Author

Reviewer #1: Thank you to the authors for this resubmission. This study still not show a clear hypothesis, which has been tested. Starting from the title, the «clinical management … using endoscopy» has for me no meaning. What I previously suggested, was the validity of the score to determine emergent endoscopy. However the score has not been developed for this. The authors showed in the course of this review a "data driven" approach, instead of an "hypothesis driven" approach, what would have been preferable. Accordingly, conclusion are not based on the data, but are derived indirectly. E.g. stating that "...patients with a JD score <3, emergency endoscopy can be avoided without any clinical disadvantage" as a conclusion is fault. In fact, 3 patients with a JD score < 3 needed emergency endoscopy. Further, the hospitalization was shorter for patients with identified bleeding source, BUT was not shorter based on the JD score (Table 2). Finally, what would have happened if patients with a JD score > 3 would have received elective endoscopy? In fact, 2 patients underwent elective endoscopy and in the group with a JD score < 3, 11 patients had an identified bleeding source necessitating clipping. This study accordingly does not determine the need for emergency lower GI endoscopy. There is still some data that are difficult to imagine, e.g. no patient had ICU care, but at least one patient received 36 (!) unit of blood (Table 2). Last but not least, the authors should be more precise on the studied population. E.g. the JD score was develop for diverticular bleeding, but the authors use "lower GI" interchangeable along the paper.

**【Answer】**

**Thank you very much for your valuable and important comments. We again revised the manuscript intensively according to your suggestions.**

**Our hypothesis was that patients with the JD score ≥3 should have emergency endoscopy and patients with the JD score** **<3 could allow elective endoscopy. We hypothesized that using the JD score in acute diverticular hemorrhage, we can effectively select the patients in whom the bleeding source can be identified when we perform emergency endoscopy (JD ≥3 group) and unselected patients can allow elective endoscopy without any clinical disadvantage when vital signs were stable (JD <3 group). For this purpose, we excluded not a few patients with the other source of bleeding and so on in this study. We had expected the former would show some clinical benefit and the latter would not show any clinical disadvantage. As a result, the former group did not provide any clinical benefit. However, more potentially ill patients (in terms of disease factors) might be included in the JD ≥3 group, and emergency endoscopy followed by hemostasis might prevent further deterioration in a patient’s condition. In the JD <3 group, elective endoscopy identified the source of bleeding in 11 of 47 patients, followed by hemostasis. There was no clinical disadvantage in this group, suggesting that the timing of elective endoscopy was not delayed. We discussed about these points on Page 22, Line351 – Page 23, Line367.**

**The JD score was developed in our previous study to identify the bleeding source efficiently in acute diverticular bleeding. To validate the JD score to determine emergency endoscopy, each patient should undergo emergency endoscopy, be categorized according to the score, and be compared regarding the bleeding source identification rate and clinical outcomes. However, these analyses are not possible with the data set.**

**When encountering a patient with suspected acute diverticular bleeding, it is an important and thorny issue to determine whether or not to perform emergency endoscopy. This is especially true for non-specialists and night shifts. We believe that it is important to assess the usefulness of the JD score to help with that decision and title changes were kept to a minimum.**

**As the reviewer pointed out, in a real clinical setting, it is more meaningful to evaluate the indication of emergency endoscopy in acute lower GI bleeding, not diverticular bleeding only. In fact, we could determine the cause of bleeding as diverticular hemorrhage, only after we performed lower GI endoscopy. So, in our next study, we are planning to extend this clinical management system to the patients with acute lower GI bleeding. We tried to distinguish diverticular bleeding from lower GI bleeding as much as possible along the manuscript and corrected the descriptions.**

**As pointed out, 3 patients with a JD score <3 needed emergency endoscopy, due to a large amount of bloody stool or unstable vital signs. Therefore, we added the description “when the vital signs were stable” on Page 3 Line 47, Page 10 Line 177, Page 22 Line 352, Page 25 Line 406, Page 27 Line 433.**

**Thanks to the suggestion of reviewer 3, we changed the statistical analysis method from Fisher’s exact test to chi-square test. Then, the number of patients who required IVR and the incidence of rebleeding at one-month post-discharge became significantly higher in the JD score ≥3 group than in the JD <3 group. We corrected Tables 2 & 3 and the descriptions in the manuscript (Page 3 Line 40-41, Page 16 Line 268-269, 274-275, Page 20 Line 309-311, Page 22 Line 348-350, Page 23 Line 362-363). We also corrected the p-values for various items in Tables 2 & 3. Anyway, emergency endoscopy did not provide any benefit in clinical outcomes of the** **JD ≥3 group. However, a significantly higher rate of bleeding source identification accompanied with hemostatic procedure might avoid serious consequences and provide clinical outcomes comparable to those in the JD <3 group. The hospitalization was shorter for patients with identified bleeding source, and we believe that to increase the pre-examination probability of bleeding source identification with the JD score was meaningful. Also, JD score might be a useful predictor of the risk of rebleeding during follow-up. We described these on Page 22 Line353- Page 23 Line 367.**

**It is an important point to see what would have happened if patients with a JD score ≥3 would have received elective endoscopy. However, it was impossible to see it in this study. As we described in the “Introduction” (Page 4 Line 73- Page 5 Line 77), there are many reports questioning the therapeutic benefits of emergency endoscopy owing to the fact that the majority of diverticular bleeding cases have spontaneous hemostasis. However, the bleeding source identification rate of these studies were low (22****－40 %) and then, we again believe that to increase the pre-test probability is useful. For a future study, a RCT study to allocate the patients with JD score ≥3 randomly to emergency endoscopy or elective endoscopy seems to be interesting.**

**As pointed out, in the JD ≥3 group one patient received 36 unit of blood transfusion and one patient received 22 unit of blood transfusion. The former patient stayed in ICU for 7 days. We apologize for not mentioning it. We should have been more precise on the studied population. We reviewed the patients’ records carefully and provided additional patient information on Page15 Line258-Page16 Line271. Also, we changed the description of patients who required IVR during hospitalization. As had been described, one patient underwent IVR for initial hemostasis after the failure of hemoclips (Page 15, Line 248-250). There were four more patients who underwent IVR for recurrent bleeding during hospitalization. There was a significant difference in the number of patients who received IVR during hospitalization. We corrected and added some items in Tables 2 & 3 and revised the descriptions on Page16 Line 268-269, Page 20 Line 309-311, Page22 Line 347-350, Page23 Line355 -Line 358, and Page25 Line 399-401)**. **This significant difference probably implies that more potentially ill patients (in terms of the disease factor) were included in the JD ≥3 group than in the JD <3 group.**

Reviewer #2: This further review of the work perhaps places it in its own specific domain. A pilot study for an initial investigation toward identifying a possible categorization of this type of patient. To accept the work as a multicenter study would have been too much. As a pilot study is the right placement of the work done.

**【Answer】**

**Thank you for your input.　As you indicated, the number of hospitals was too small to be considered as a multicenter study. We have removed the word "multicenter". We will continue to accumulate cases and strive to develop our research with better study designs.**

Reviewer #3: The utility of the JD score devised for patients with diverticular bleeding was evaluated. The primary and secondary endpoints were the bleeding source identification rate and clinical outcomes. The rate of bleeding source identification was significantly higher in the JD ≥3 group than in the JD <3 group. The groups did not differ significantly with respect to clinical outcomes.

Minor revisions:

1- Line 191: State the statistical testing method which achieves 80% power.

2- Line 204: Use a lower case p for “p-values”.

3- For some comparisons of proportions, chi-square tests may be more powerful than Fisher’s exact tests, considering the expected sample sizes.

**【Answer】**

**Thank you very much for your valuable and useful comments.**

**1- The statistical testing method was paired *t*-test. We added the description on Page11, Line 191.**

**2- We have corrected the notation of p-values.**

**3-　Thank you for your very useful suggestions. We have changed the statistical method to a chi-square test. As a result, some significant differences have become apparent or disappeared. In Table 2, "Re-bleeding after 1 month," a significant difference became apparent (p = 0.079 to 0.028). In Table 3, "Dialysis," a significant difference disappeared (P=0.046 to 0.098). Accordingly, some of the text has been changed (Page3 Line 40, Page16 Line 273-275, Page 20 Line 309-311, Page 22 Line348-350, Page 23 Line362-363). This significant difference has shown a new possibility of predicting rebleeding after 1 month by JD score and we added the description (Page 23, Line 363-367).**

**.**
